# Cesarean section after laparoscopic hysterosacropexy with Richardson’s lateral repair and Burch operation—Case report

**DOI:** 10.1016/j.ijscr.2019.05.039

**Published:** 2019-05-29

**Authors:** Paweł Szymanowski, Wioletta Katarzyna Szepieniec, Krzysztof Stuwczyński, Paweł Gruszecki

**Affiliations:** Andrzej Frycz Modrzewski Krakow University, Department of Gynecology and Urogynecology, ul. Kostrzewskiego 47, 30-437 Krakow, Poland

**Keywords:** Laparoscopic hysteropexy, Case report, Pregnancy, Delivery

## Abstract

**Introduction:**

Pelvic floor disorders are common also in young patient population. Their quality of life is lessened because of their condition, but they would also like to avoid any complicated operations. The minimal invasive procedures and fertility sparing are very important.

**Case presentation:**

32 year old caucasian female (BMI: 22,6) was admitted to our clinic in August 2016 with the following symptoms: the feeling of a foreign body in the vagina, prolapse of the bladder and stage II stress urinary incontinence. She had 2 vaginal deliveries. A gynecological examination of the pelvic organ prolapse was classified as follows: prolapse of the uterus POP-Q II, and a cystocele with a lateral defect POP-Q III, after reposition of uterus cystocele POP-Q II, urethrocele, positive cough test.

She underwent a laparoscopic hysteropexy with lateral repair and Burch operation. Effect of the operation was satisfied. It revealed an improvement in the QoL-Questionnaire PIFQ-7.

Pregnancy in 2018 was delivered by cesarean section. The effect of the prolapse operation was not affected and the quality of life maintained.

**Conclusion:**

Laparoscopic hysteropexy should be a standard procedure for the repair of a level I defect in young patients, particularly as a fertility sparing procedure.

## Highlights

• Uterus preserving therapy is an optimal therapy for young patients.
• Using of a small mesh can reduce the use of large meshes in pelvic floor surgery.
• Pregnancy and delivery after hysterosacropexy does not affect the effect of the operation.
• Quality of life is improved.

## 1 Introduction

Younger patients with pelvic floor disorders are an increasing sight in the everyday practice of gynecology. This group of patients, however, represents an intriguing problem from a therapeutic point of view. Their quality of life is lessened because of their condition, but they would also like to avoid any complicated operations.

Due to this, laparoscopic operative techniques are often viewed as more favorable than other types of surgery because of the reduced invasiveness of the operation. The effects of pelvic floor disorders can be, among others, a decreased quality of sexual life and an adverse effect on their ability to have children. Many young patients plan to have children and as there is no significant pathology of the genitals they desire a less invasive operation which does not cause any long-term, negative changes to their body.

Bearing this in mind it would be beneficial to take a new look at surgical procedures concerning level I defects. A level I defect is caused by a lesion of the sacrouterine ligaments and the proper treatment of this defect is not the excision of the uterus. A hysterectomy, which in many cases is still used to treat this problem, is not a causal therapy, meaning that it does not directly address the source of the problem, and it can also lead to a very high risk of vaginal vault prolapse [1].

The case presented here is of a 32 year old patient who successfully underwent a minimally invasive laparoscopic bilateral hysterosacropexy, which preserved the uterus, combined with lateral paravaginal repair and the Burch operation. After undergoing this operation she became pregnant and subsequently delivered by cesarean section. The pregnancy and cesarean section proceeded without any complications and did not affect the correction of the apical defect which had been achieved by the laparoscopic hysterosacropexy with paravaginal repair and the Burch operation.

This work has been reported in accordance with the SCARE criteria [2].

## 2 Presentation of the case

A 32 year old caucasian female (BMI: 22,6) was admitted to our clinic in August 2016 with the following symptoms: the feeling of a foreign body in the vagina, prolapse of the bladder and stage II stress urinary incontinence. The anamnesis showed the following: regular menstruation and two vaginal deliveries (in 2011 - female newborn weight 4000 g, length 53 cm and in 2015 a female newborn weight 4490 g, length 53 cm).

A gynecological examination of the pelvic organ prolapse was classified as follows: prolapse of the uterus POP-Q II, and a cystocele with a lateral defect POP-Q III, after reposition of uterus cystocele POP-Q II, urethrocele, positive cough test. A transvaginal ultrasound showed a normal retroflected uterus, no pathologies in both ovaries, no urine retention in the bladder after miction and no urine retention in both kidneys.

Because of the young age of the patient, a lack of pathologies of the genitals and the patient’s wish to maintain her fertility a laparoscopic operation was indicated: laparoscopic bilateral hysterosacropexy with Richardson’s paravaginal repair and the Burch operation.

This operation was performed on 30.08.2016 according to internal clinical standards.

On the day of the surgery, the patient was placed under general endotracheal anesthesia in the lithotomy position. After routine preparation, a sterile catheter was placed in the bladder and a bullet forceps was placed upon the cervix in order to manipulate the uterus during the procedure. A 10 mm trocar was inserted directly into an incision in the umbilical crease without the use of a Veress needle and carbon dioxide was insufflated into the peritoneum. After insertion of a 30° camera, three more trocars were placed into the lower abdomen 10 mm and 5 mm on the left side and 5 mm trocar on the right side of the patient. Inspection of the genital organs revealed no pathologies and the other organs showed no macroscopic changes. The patient was placed in the Trendelenburg position to ensure visibility of the whole pelvis.

**Image 1:**
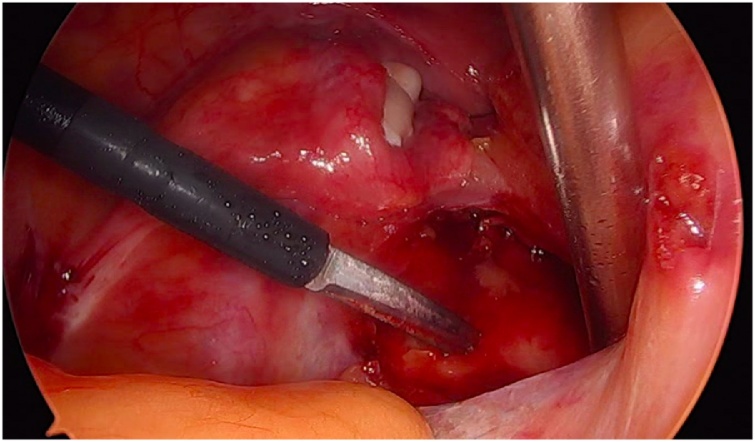
Peritoneal incision at the promontory level on the left side.

**Image 2:**
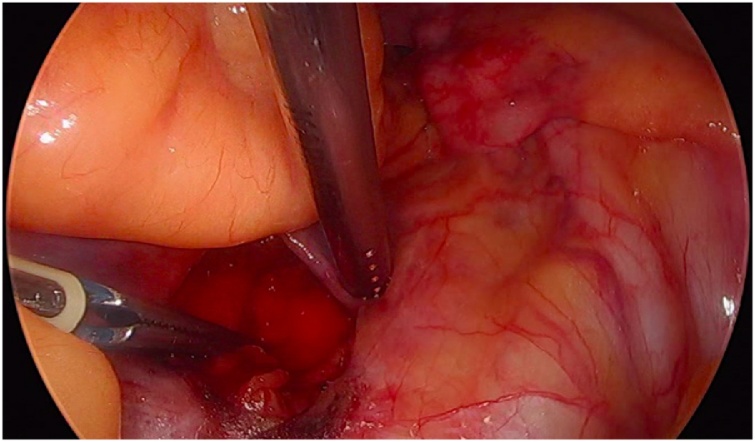
Peritoneal incision at the promontory level on the right side.

**Image 3:**
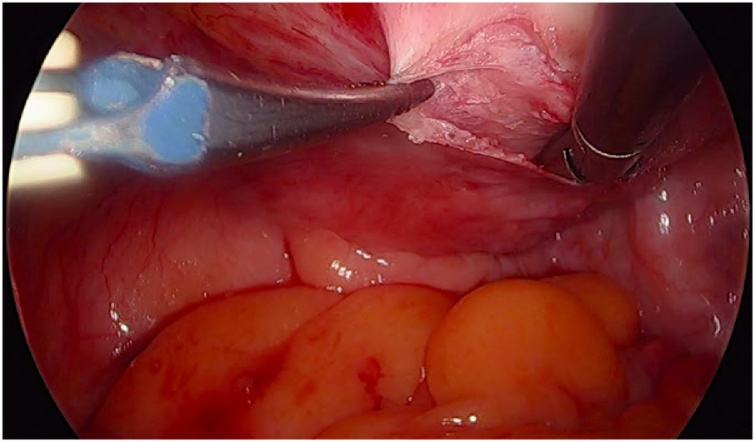
Incision at the posterior wall of the cervix.

**Image 4:**
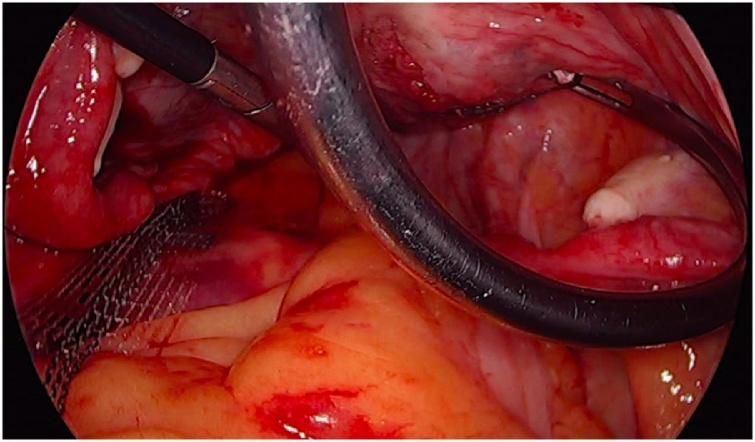
Using of the Neyemeyer Helix.

**Image 5:**
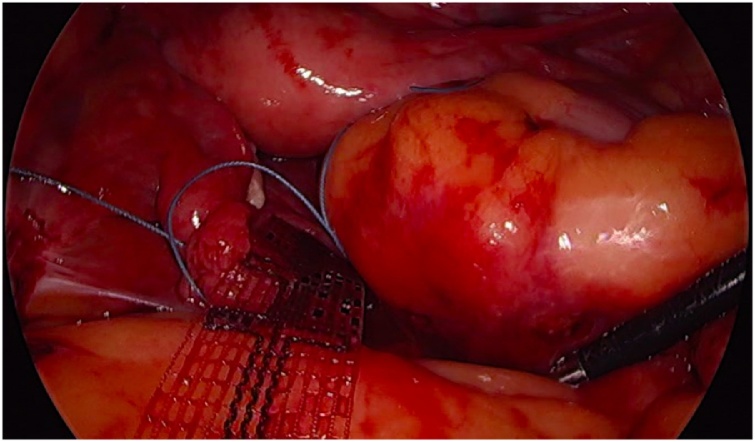
MESH-tape used for this procedure.

**Image 6:**
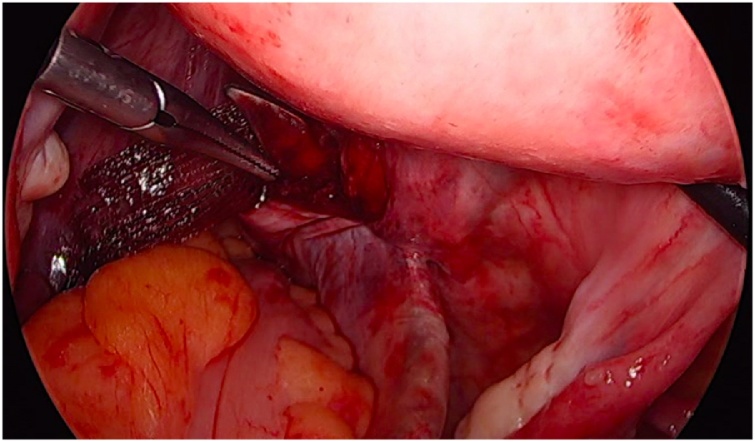
Tunneling of a canal with Neymeyer Helix.

**Image 7:**
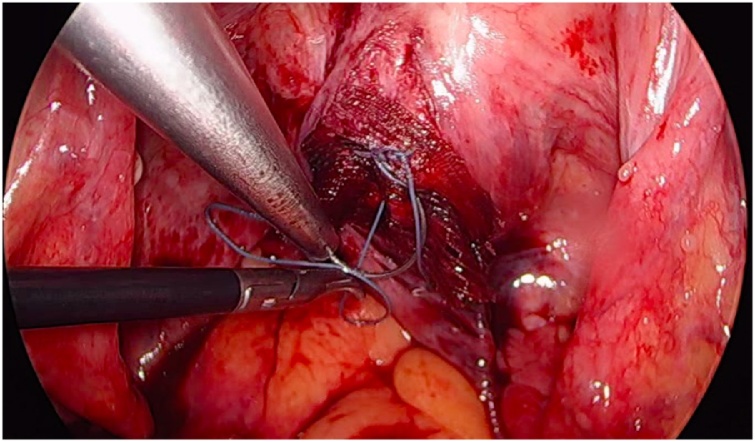
Suture of the MESH to the posterior wall of the cervix.

Initially, the left ureter was found and the peritoneum was opened over the anterior longitudinal ligament of the spine at the promontory level [Image 1]. The same procedure was then performed on the right side [Image 2]. The anterior longitudinal ligament of the spine was exposed on both sides without injuring the inferior hypogastric plexus or the median sacral artery. Next, an incision was made on the parietal peritoneum on the dorsal wall of the cervix uteri and it was then partially separated from the cervix [Image 3]. Using a Neymeyer Helix [Image 4], which was inserted directly through the abdominal wall, a retroperitoneal tunnel was formed between the cervix uteri and the peritoneal incision on the left side following the anatomical location of the left uterosacral ligament using an alloplastic tape, DynaMesh®-CESA, which is monofilament polyvinyl difluoride for the hysterosacropexy measuring 20 x 1 cm and then the proximal end of the graft was pulled through the retroperitoneal tunnel [Image 5]. After that a retroperitoneal tunnel was formed on the right side between the incision of the peritoneum at the promontory level and the cervix uteri, following the anatomical location of the right uterosacral ligament [Image 6]. The proximal end of the graft was pulled through the retroperitoneal tunnel on the right side. Next, the distal end of the mesh was anchored to the cervix with non-absorbable interrupted sutures 2-0 [Image 7]. The proximal end of the graft was attached to both the right and left side of the longitudinal anterius ligament on the sacral spine with the same type of sutures. All excessive mesh was removed so that approximately 8 cm of mesh remained. The peritoneum over the sutured mesh was carefully closed with absorbable Vicryl 2-0 sutures to prevent the intestines from coming into contact with the graft.

The Burch operation and lateral repair were both performed in the standard manner. After preparation of the bladder and the prevesical space 2 non resorbable 2-0 sutures were placed on both sides of the vagina and sutured to the pectineal ligament. The Burch operation was also performed with the same material placed paraurethral on each side of the vagina. Suturing should be done without tension to avoid any hypercorrection.

Hemostasis was ensured, all instruments were removed from the abdomen under visual control and the incisions were closed in the standard fashion.

The procedure was completed without any complications. On the day of discharge, the postoperative site showed no defect at level I, while presenting a cystocele POP Q I and a rectocele POP Q I at level II.

The operation resulted in a correction of the level I defect to POP-Q 0. Follow-up examinations were performed after 2 weeks and also after 6 months postoperatively. The patient had no further complaints of dysuria or urinary retention and there was no evidence of urinary incontinence.

**Image 8:**
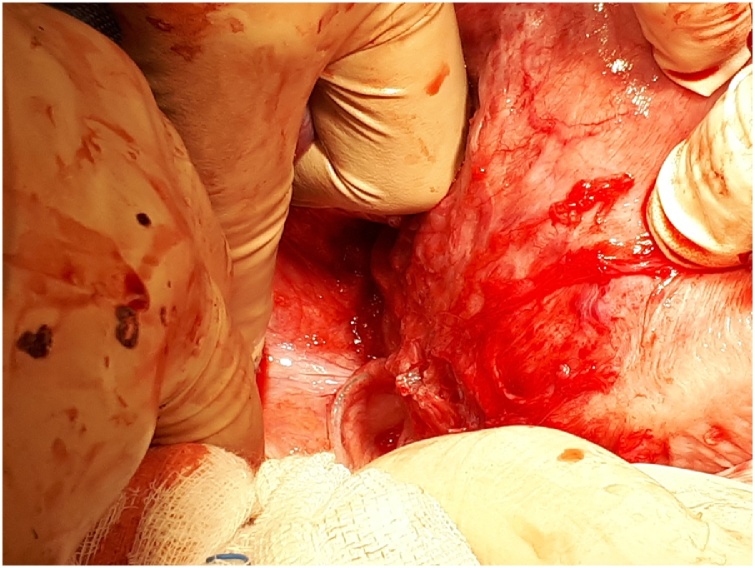
Tape in situ by the cesarean section – left side.

**Image 9:**
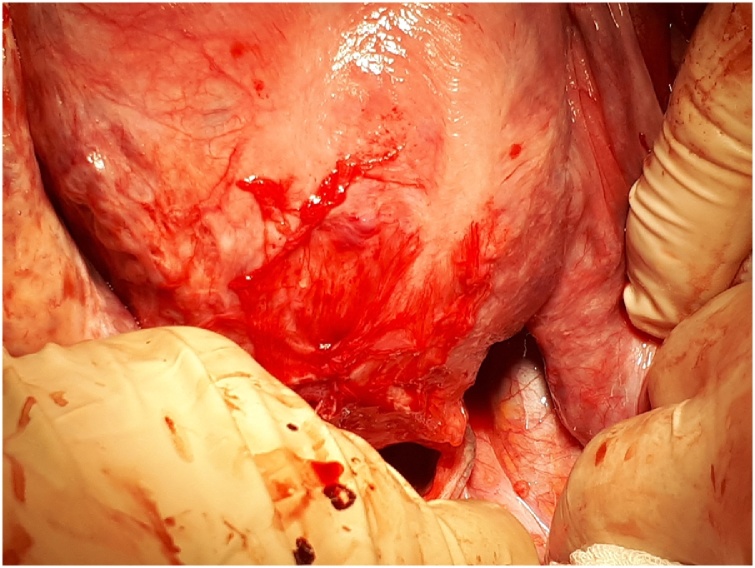
Tape in situ by the cesarean section – right side.

The patient was subsequently registered in September 2017 as an outpatient in the 7^th^ week of pregnancy with no complaints of urinary incontinence and a gynecological examination revealed no changes at either level I or II when compared to the postoperative state. The pregnancy was physiological and the agreed-upon delivery method was cesarean section with the birth taking place in April 2018. (female newborn weighing 3220 g and 54 cm in length). The tape was observed in situ [Images 8 and 9]. Both the birth and postoperative observation time proceeded without any complications. A follow-up examination 6 weeks postpartum was normal and the last follow-up in February 2019 also revealed no problems.

The patient does not complaint of any discomfort, pain or urge incontinence. The follow-up examination revealed no defect at the level I and didn’t show negative influence on level II in anterior compartment. The QoL-Questionnaire PIFQ-7 revealed remarkable improvement - 73 pts before surgery, 2 pts after hysterosacropexy with lateral repair and 3 pts after the cesarean section.

## 3 Discussion

3–6% of patients have symptoms of pelvic organ prolapse (POP) and when undergoing a vaginal examination the number rises to 50% [3].

After searching the medical databases, such as Medline or Cochrane, no sufficient data about urinary incontinence and pelvic organ prolapse for pregnant and postpartum women was found [4].

The surgical treatment of young patients should be adjusted according to their wishes and symptoms grade with surgery not being entirely dependent on issues of family planning, thus freeing the physician to treat the patient properly, but also allow the patient to have children later if so desired.

The factors which are important are the method in which the pregnancy is followed up, the patient’s lifestyle and behavior, and physiotherapy for the protection of the pelvic floor muscles.

The most common risk factor for pelvic floor disorders is birth by vaginal delivery, or more accurately, operative vaginal delivery, such as by forceps, which increases the risk of sphincter injury and POP [5].

Birth by cesarean section has a protective value concerning stress urinary incontinence, overactive bladder and pelvic organ prolapse. Dietz et al. show that the sonographic parameters of pelvic organ descent and pelvic floor muscles were not statistically affected after subsequent cesarean sections under the condition that the first delivery was also a cesarean section [6].

The authors suggest that delivery by cesarean section is indicated in patients who have had pelvic floor symptoms after a vaginal delivery and underwent surgery for POP.

The authors also suggest that conservative treatment of POP in young patients is not a standard management. In a paper by Lowenstein et al. in older patients a significant positive effect as a result of the POP correction meant that women had a better self-perceived body image and sexual function. Pessary treatment does not improve these parameters [7].

The surgical treatment of the pelvic floor in cases of POP can substantially affect the quality of sexual life and self-esteem of the patients. Performing a hysterectomy, on the other hand, does not positively affect either of these. In a paper by Detollenaere there was no statistically significant difference in overall sexual functioning between sacrospinous hysteropexy and vaginal hysterectomy with suspension of the uterosacral ligaments after a follow-up period of 24 months [8]. In another study, Glavind showed a significant improvement after different POP operations using native tissue repair [9].

It seems to be a more and more common assertion by various authors that the optimum solution in cases of POP is surgery which leaves the uterus intact. It is important for the fertility of patients and also has an impact on possible future complications.

It is interesting that in a paper by Gutman which compared sacral colpopexy after a hysterectomy and sacral hysteropexy the anatomical outcomes were the same but the risk of mesh exposure is five times higher for the former, independent of the technique - open or laparoscopy [10].

An analysis conducted by Barber concerning surgery for vaginal prolapse shows that abdominal sacral colpopexy in comparison to sacrospinous colpopexy has a lower risk of stress urinary incontinence and dyspareunia. This analysis also revealed less loss of blood and hospitalization time for laparoscopic operations. In another analysis Barber shows better outcomes of abdominal sacral colpopexy by using polypropylene mesh instead of fascia lata.

2 RCT [11,12] showed the same success rate of a vaginal hysterectomy with uterosacral ligament suspension as with sacrospinous hysteropexy for apical compartment support. Both RCTs also revealed a shorter hospitalization time and quicker return to work after sacrospinous hysteropexy.

The FDA issued a warning about MESH in 2011 which is an indication the seriousness of the problem with alloplastic materials [13]. Alloplastic material in the human body can lead to symptoms like pain, discomfort, or erosion of the vaginal wall and these in turn may cause vaginal bleeding and dyspareunia.

In the hysterosacropexy procedure only a narrow tape is used. The use of this kind of tape reduces the amount of alloplastic material. In the lateral repair and Burch procedure only sutures are used thus making the procedure being described in this paper operation compatible with current indications for the reduction of alloplastic materials in urogynecology.

The authors would like to emphasize the lack of data concerning surgical procedures in the treatment of pelvic floor disorders for sparing the fertility of the patient. Laparoscopic hysteropexy can be an effective method for young patients who would like to possibly have children in the future. The current paper shows the high probability of the sustained effectiveness of this procedure even after delivery. The authors suggest the use of a cesarean section is to minimize the relapse of the defect after the pregnancy and also after other pelvic floor operations.

## 4 Conclusion

Laparoscopic hysteropexy is a viable option for the correction of a level I defect and in fact it should be the preferred method instead of uterosacral ligament colpopexy because of its better therapeutic effect. Vaginal meshes should not be used in young patients because of a lack of scientific data from long-term studies concerning their use.

Laparoscopic hysteropexy should be a standard procedure for the repair of a level I defect in young patients, particularly as a fertility sparing procedure. Also lateral repair in case of concomitant lateral level II defect seems to be a good therapeutic option for this group of patient. Pregnancy after this procedure is not contraindicated as long as there is special prenatal care and the method of delivery is correct. Currently there is a lack of evidence for uterus sparing procedures in the treatment of a level I defect but among young patients this method seems to have no negative effect on fertility.

## Conflicts of interest

No conflict of interest for all authors.

## Sources of funding

No payment was authorized by any source.

## Ethical approval

No specific ethical approval was necessary, because this case report is related to an operative procedure necessary to maintain the patient’s health. Specific informed consent and authorization for publication of anonymous data was obtained from the patient.

## Consent

Written informed consent was obtained from the patient for the publication of this case report and accompanying images. On request, a copy of the written consent is available for review by the Editor-in-Chief of this journal.

## Author’s contribution

Each author contributed to diagnosis, treatment, and postoperative follow-up of patient. Specific recording of pathologic data and an adequate review of literature was performed by each author.

## Registration of research studies

Each author contributed to diagnosis, treatment, and postoperative follow-up of patient. Specific recording of pathologic data and an adequate review of literature was performed by each author.

## Guarantor

Pawel Szymanowski MD, PhD.

## Provenance and peer review

Not commissioned, externally peer-reviewed.
